# Assessing the Antitumor Potential of Variants of the Extracellular Carbohydrate Polymer from *Synechocystis* Δ*sigF* Mutant

**DOI:** 10.3390/polym15061382

**Published:** 2023-03-10

**Authors:** Rita Mota, Raquel T. Lima, Carlos Flores, Juliana F. Silva, Beatriz Cruz, Bárbara Alves, Marta T. Pinto, Alessandra Adessi, Sara B. Pereira, Roberto De Philippis, Paula Soares, Paula Tamagnini

**Affiliations:** 1 i3S - Instituto de Investigação e Inovação em Saúde, Universidade do Porto, Rua Alfredo Allen 208, 4200-135 Porto, Portugal; rita.mota@ibmc.up.pt (R.M.); rlima@ipatimup.pt (R.T.L.); carloseduardof1990@gmail.com (C.F.); jusilva.fm@gmail.com (J.F.S.); beatrizscruz0798@gmail.com (B.C.); barbaracvalves@gmail.com (B.A.); mtpinto@ipatimup.pt (M.T.P.); sarap@ibmc.up.pt (S.B.P.); psoares@ipatimup.pt (P.S.); 2 IBMC - Instituto de Biologia Molecular e Celular, Universidade do Porto, Rua Alfredo Allen 208, 4200-135 Porto, Portugal; 3 IPATIMUP - Institute of Molecular Pathology and Immunology of the University of Porto, Rua Alfredo Allen 208, 4200-135 Porto, Portugal; 4 FMUP - Department of Pathology, Faculty of Medicine, University of Porto, Alameda Prof. Hernâni Monteiro, 4200-319 Porto, Portugal; 5 ICBAS - Instituto de Ciências Biomédicas Abel Salazar, Universidade do Porto, Rua de Jorge Viterbo Ferreira 228, 4050-313 Porto, Portugal; 6 FCUP - Departamento de Biologia, Faculdade de Ciências, Universidade do Porto, Rua do Campo Alegre, s/n, 4169-007 Porto, Portugal; 7 School of Allied Health Sciences of Polytechnic Institute of Porto, Rua Dr. António Bernardino de Almeida 400, 4200-072 Porto, Portugal; 8 DAGRI - Department of Agriculture, Food, Environment and Forestry, University of Florence, Via Maragliano 77, 50144 Firenze, Italy; alessandra.adessi@unifi.it (A.A.); roberto.dephilippis@unifi.it (R.D.P.)

**Keywords:** antitumor activity, CAM assay, cancer, carbohydrate polymer, cyanobacteria, *Synechocystis*

## Abstract

Cancer is a leading cause of death worldwide with a huge societal and economic impact. Clinically effective and less expensive anticancer agents derived from natural sources can help to overcome limitations and negative side effects of chemotherapy and radiotherapy. Previously, we showed that the extracellular carbohydrate polymer of a *Synechocystis* Δ*sigF* overproducing mutant displayed a strong antitumor activity towards several human tumor cell lines, by inducing high levels of apoptosis through p53 and caspase-3 activation. Here, the Δ*sigF* polymer was manipulated to obtain variants that were tested in a human melanoma (Mewo) cell line. Our results demonstrated that high molecular mass fractions were important for the polymer bioactivity, and that the reduction of the peptide content generated a variant with enhanced *in vitro* antitumor activity. This variant, and the original Δ*sigF* polymer, were further tested *in vivo* using the chick chorioallantoic membrane (CAM) assay. Both polymers significantly decreased xenografted CAM tumor growth and affected tumor morphology, by promoting less compact tumors, validating their antitumor potential *in vivo*. This work contributes with strategies for the design and testing tailored cyanobacterial extracellular polymers and further strengths the relevance of evaluating this type of polymers for biotechnological/biomedical applications.

## 1. Introduction

Cancer is a leading cause of death worldwide, with an estimation of 19.3 million new cases and almost 10 million deaths in 2020, thus having a huge societal impact and economical relevance. Based on the GLOBOCAN project in 2020, these numbers are expected to grow to 28.4 million new cancer cases in 2040, a 47% rise from 2020 [1]. These projections increase the demand of early cancer detection and the development of advanced and innovative therapies. Due to the limitations and negative side effects of chemotherapy and radiotherapy, researchers are focused on finding alternative therapeutics that are clinically effective and less expensive, namely anticancer agents from natural sources [2,3,4]. In this context, biopolymers of polysaccharidic nature embody promising therapeutic alternatives, by preventing tumor development and inhibiting proliferation, invasion, adhesion, metastization, and/or angiogenesis [3,5]. These biopolymers can also act in combination with conventional anticancer drugs, improving their activity through the enhancement of tumor sensitivity and patient immune response [3,6]. The molecular mechanisms underlying the biopolymers’ antitumor effects include cell cycle arrest, activation of the mitochondrial-mediated apoptotic pathway, production and activation of nitric oxide pathway or immunomodulatory pathways [7]. While some biopolymers may act through defined pathways, for others the mechanism of action is still not clear, but may include production of reactive oxygen species, as well as inhibition of proteins involved in angiogenesis and metastization, among other processes [3]. In recent years, cyanobacteria have emerged as a promising source of bioactive polysaccharides, since most strains produce extracellular polymeric substances (EPS), mainly composed by heteropolysaccharides, with a distinctive set of desirable characteristics, including a (i) large number of different monosaccharides, allowing a wide range of structural rearrangements, (ii) strong anionic character due to the presence of uronic acids and sulfate groups and (iii) presence of deoxyhexoses and peptides that contribute to hydrophobicity and, thus, amphiphilic behavior [8,9]. These peculiar features prompted significant advances on their characterization [10,11,12] and validation of their potential for biomedical applications [13,14,15,16,17,18,19]. However, studies on the antitumor potential of cyanobacterial EPS are still scarce and mainly based on tumor cell lines [20,21,22,23,24,25], with few *in vivo* studies using tumor xenograft mice models [26].

Previously, we showed that a sigma factor mutant (Δ*sigF*) of the model unicellular cyanobacterium *Synechocystis* sp. PCC 6803 had the secretion mechanisms significantly altered, being able to release up to four-fold more polysaccharides (RPS - released polysaccharides) compared to the wild-type [27,28]. These carbohydrate polymers displayed a strong antitumor activity towards human tumor cell lines, namely melanoma (Mewo), thyroid carcinoma (8505C) and ovarian carcinoma (A2780), being the Δ*sigF* polymer easier to isolate and recover from the culture medium and more potent than the one from the wild-type [20]. We also showed that the antitumor effect of the Δ*sigF* polymer was associated with the induction of apoptotic pathways via upregulation of p53 protein levels and caspase-3 activation [20]. Importantly, the Δ*sigF* polymer induced one of the highest levels of apoptosis (~40%) observed among natural polymers [20,23,29,30]. Despite these results, it is still unclear which are the polymer features/constituents that mostly contribute to its antitumor activity.

The major aim of this study was to manipulate the *Synechocystis*’ Δ*sigF* polymer in order to obtain variants with increased antitumor activity (*in vitro* and/or *in vivo*), gaining insight into key features that contribute to the polymer bioactivity.

## 2. Materials and Methods

### 2.1. Cyanobacterial Strain and Culture Conditions

*Synechocystis* sp. PCC 6803 Δ*sigF* [27,28] was grown in Erlenmeyer flasks with BG11 medium [31] at 30 °C under 12 h light (50 μE/m^2^/s)/12 h dark regimen, with orbital shaking at 150 rpm. 

The Δ*sigF* mutant was maintained in BG11 medium supplemented with kanamycin (100 μg/mL), while the experiments were performed in the absence of selective pressure.

### 2.2. Polymer Isolation 

The polymer was isolated according to [32,33]. Briefly, the *Synechocystis* Δ*sigF* cultures were dialyzed (12–14 kDa of molecular weight cut-off; Medicell International, London, UK) against a minimum of 10 volumes of deionized water for 48 h with continuous stirring. Then, the cultures were centrifuged at 20,000× *g* for 25 min at 8 °C, and 2 volumes of 96% ethanol were added to the supernatant. After an incubation at 4 °C overnight, the suspension was centrifuged at 20,000× *g* for 25 min at 6 °C, and the supernatant was discarded. The pellet was resuspended in 1 mL of autoclaved type II water and lyophilized. The dried polymer was stored at room temperature (RT) until further use.

### 2.3. Polymer Hydrolysis and Molecular Mass Analysis

Hydrolysis of the polymers was performed with either 2 M trifluoroacetic acid (TFA) (VWR Chemicals, Radnor, PA, USA) at 120 °C or in 2 M hydrochloric acid (HCl) (Merk, Darmstadt, Germany) at 100 °C for 10 min, 25 min, 45 min, 1 h, or 2 h. After hydrolysis, the samples were evaporated two times, and the polymers resuspended in ultrapure water before being analyzed by size exclusion chromatography (SEC).

The apparent molecular masses (hereafter referred to as MM) were determined according to a previously reported method [34], with some changes. Briefly, samples were dissolved in high-performance liquid chromatography (HPLC) grade water at a concentration of 5 mg/mL and analyzed using a Varian ProStar HPLC chromatograph (Varian, Palo Alto, CA, USA) equipped with a 355 RI (refractive index) detector and two columns for SEC, Polysep-GFC-P 6000 and 4000 (Phenomenex, Torrance, CA, USA) connected in series. The analyses were performed with runs of 70 min and with HPLC grade water as eluent at a flow rate of 0.4 mL/min, using Dextran (Sigma–Aldrich, Burlington, MA, USA) at different MM (2000 kDa, 1100 kDa, 410 kDa, 150 kDa, and 50 kDa) and saccharose (0.34 kDa) as standards.

### 2.4. Quantification of Sulfate and Peptide Contents

For sulfate quantification, lyophilized RPS were hydrolyzed in 2 M HCl at 100 °C for 10 min to 2 h, centrifuged after cooling, and the supernatant was analyzed by ion-exchange chromatography [35,36]. The analysis was performed using a Dionex ICS-2500 system chromatograph equipped with a continuously regenerated anion-trap column, a continuous anionic self-regenerating suppressor, a conductivity detector (ED50), an IonPac PA11 4 × 250 mm column (Dionex, Sunnyvale, CA, USA), and a reagent-free Dionex system producing high-purity 50 mM KOH at a flow rate of 2 mL/min. Sulfate solutions (1 to 10 mg/L, Fluka, Switzerland) were used as standards.

To evaluate the peptide content, the lyophilized polymer was resuspended in deionized water and quantification was performed by the Lowry method [37].

### 2.5. Peptide Removal and Analysis

Peptide removal was performed by trichloroacetic acid (TCA) (Fisher Bioreagents, Bremen, Germany) precipitation [35,36]. Briefly, an aqueous solution polymer (5 mg/mL) was incubated with 15% TCA for 15 or 30 min at room temperature. After incubation, the mixture was centrifuged at 11,000× *g* for 20 min at 4 °C. The supernatant was dialyzed against a minimum of 10 volumes of deionized water for 48 h with continuous stirring and lyophilized. The precipitation efficiency was assessed measuring the peptide content in the polymer suspension before and after TCA treatment using the Lowry method [37].

### 2.6. Human Tumor Cell Lines and Culture Conditions

The human melanoma (Mewo) cell line (kindly given by Prof. Marc Mareel, Department of Radiotherapy and Nuclear Medicine, Ghent University Hospital, Belgium) [38] was maintained in DMEM culture medium with stable glutamine (Capricorn Scientific, Ebsdorfergrund, Germany), supplemented with 10% fetal bovine serum (FBS, GIBCO, Invitrogen, UK), 1x penicillin/streptomycin (Biowest, Nuaillé, France), and 1.25 μg/mL amphotericin B (Corning, NY, USA). Cells were maintained in a humidified incubator at 37 °C with 5% CO_2_. Cell lines were authenticated following genotyping at i3S Genomics Core Facility (Porto, Portugal) using the PowerPlex^®^ 16 HS System (Promega, Madison, WI, USA) and confirmation with the DNA profiles available at ATCC and ECACC STR profiles database.

### 2.7. Cell Viability Assays

Mewo cells were plated on 96-well plates at 1 × 10^4^ cells/well and allowed to adhere for 24 h at 37 °C. Cells were then treated for 24 and 48 h with: (i) supplemented media (Blank), (ii) Δ*sigF* polymer variants (resuspended in non-supplemented media) at 0.7, 1, and 1.5 mg/mL, or iii) with polymer vehicle as control (consisting of supplemented media containing the equivalent amount of non-supplemented medium used in the polymer treatments). To assess cell viability after treatment, a PrestoBlue^TM^ cell viability assay was carried out as previously described [39]. Briefly, cells were washed three times with the respective non-supplemented medium and further incubated for 45 min with 10% PrestoBlue^TM^ reagent (Life Technologies, Carlsbad, OR, USA) in supplemented medium. Fluorescence was measured (excitation 560 nm; emission 590 nm) on a Synergy HT Multi-Mode Microplate Reader (BioTek Instruments Inc., Santa Clara, CA, USA). All samples and controls were analyzed with four biological and five technical replicates. Cellular viability was determined by analyzing the fluorescence values of each sample as a percentage in relation to control samples (cells treated with medium only), after removing the background values.

### 2.8. CAM Assay

The effect of polymers on tumor growth and angiogenic activity was evaluated *in vivo* using the chick chorioallantoic membrane (CAM) assay [40,41,42]. Fertilized chick eggs (*Gallus gallus*) were incubated horizontally at 37 °C in a humidified atmosphere and referred to the embryonic development day 0 (EDD0). On EDD3, eggs were prepared (window opening) to allow detachment of the developing CAM from the shell. On EDD9, CAMs were inoculated with 1 × 10^6^ exponentially growing Mewo cells resuspended in Matrigel (1:1) together with (i) 0.7 mg/ml of Δ*sigF* polymer, (ii) its variant obtained after 15 min of TCA treatment (Δ*sigF*.pep-), or iii) with their vehicle solvent (DMEM), into a 5-mm silicone ring under sterile conditions. After resealing, eggs were returned to the incubator. At EDD13, CAMs were fixed in 10% neutral-buffered formalin, the ring was removed, and CAMs were excised from the embryos and photographed *ex ovo* under a stereoscope at 20× magnification (Olympus, Tokyo, Japan, SZX16 coupled with a DP71 camera). The number of new vessels growing towards the inoculation site, delimited by the ring mark were counted (less than 20 μm in diameter) and the area of the tumors (dense areas) were determined using the “Cell A Olympus” program, as described previously [40,41,42].

### 2.9. Tumor Histology and Immunohistochemical Analysis

Excised CAMs (fixed in 10% neutral-buffered formalin) were processed for paraffin-embedding. Serial 4-µm-thick sections were cut for hematoxylin and eosin (H&E) stain and for immunohistochemistry evaluation of Ki-67 and cleaved caspase-3. Briefly, for immunohistochemistry, after deparaffinization and rehydration, samples were subjected to heat-induced antigen retrieval in citrate buffer, pH 6 (Richard-Allan Scientific, Kalamazoo, MI, USA) for Ki-67 or in Epitope Retrieval solution pH 9.0 (Novocastra; Leica Biosystems, Wetzlar, Germany), for cleaved caspase 3 evaluation, for 45 min at 90 °C in a steamer. Following blocking of endogenous peroxidase activity and of non-specific binding with Ultravision Hydrogen Peroxide Block and Protein Block reagents (Thermo Scientific, CA, USA), respectively, sections were incubated with Ki-67 antibody (ref. M7240, 1:200, DAKO, Glostrup, Denmark) for 30 min at RT or with cleaved-caspase 3 antibody (ref. 9664, 1:1000, Cell Signaling, Danvers, MA, USA) overnight at 4 °C. Signal amplification was carried out with Primary Antibody Amplifier incubation, followed by HRP polymer (UltraVision Quanto Detection System HRP; Thermo Scientific, CA, USA). Detection was performed using 3,3′ Diaminobenzidine (DAB Quanto Chromogen and Substrate; Thermo Scientific, CA, USA) and counterstaining with Mayer’s Haematoxylin (Thermo Scientific, CA, USA). Tonsil tissue sections were used as a positive control for staining. Negative controls (in which the primary antibody was omitted) were also included. Slides were evaluated using the light microscope Olympus DP 25 Camera coupled to the Cell B Olympus software (Tokyo, Japan). The percentage of Ki-67 positive stained cells was determined by evaluating at least 2900 tumor cells per slide. 

### 2.10. Statistical Analysis

Data were plotted and statistically analyzed using GraphPad Prism version 9.0 (GraphPad Software, San Diego, CA, USA) using an analysis of variance (ANOVA), followed by Bonferroni’s multiple comparisons test. Regarding the CAM assays, statistical analysis was performed using one-way ANOVA test with IBM SPSS statistics.

## 3. Results and Discussion

We previously demonstrated that the extracellular carbohydrate polymers released by the unicellular cyanobacterium *Synechocystis* sp. PCC 6803 and its Δ*sigF* mutant exhibited a strong antitumor activity against several human tumor cell lines, with the latter being more effective [20]. The present study goes a step forward by manipulating several features of the Δ*sigF* polymer to obtain variants, which were tested *in vitro* in the human melanoma (Mewo) cell line. The most promising variant was then evaluated *in vivo*, using the chick embryo chorioallantoic membrane (CAM) model.

### 3.1. Polymer Hydrolysis and Evaluation of Antitumor Activity In Vitro

**Figure 1:**
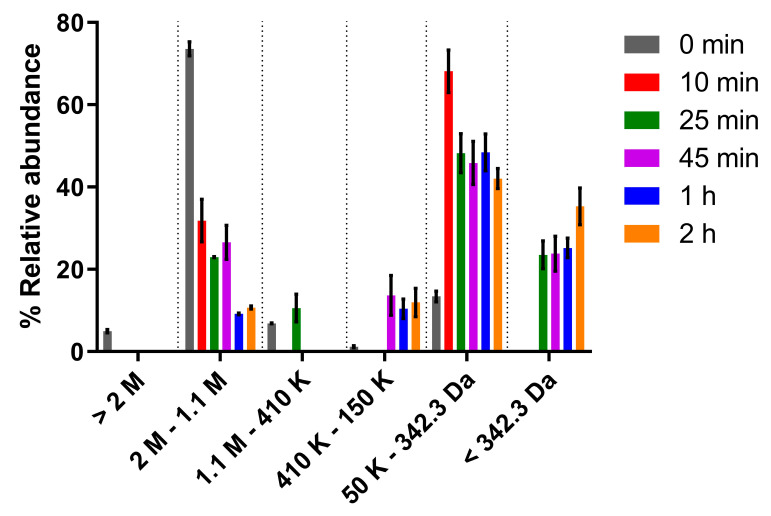
Molecular mass distribution of *Synechocystis* Δ*sigF* polymer before and after hydrolysis with trifluoroacetic acid (TFA) for different time periods. Measurements were made using size exclusion chromatography (SEC). Results are represented as mean ± STD of three biological and three technical replicates.

Since previous studies reported a correlation between the molecular mass (MM) of algal polysaccharides and their antitumor activity [43,44,45], modification of the MM distribution of *Synechocystis* Δ*sigF* polymer was firstly achieved through trifluoroacetic acid (TFA) hydrolysis (Figure 1). A gradual increase of the abundance of lower MM fractions was observed by increasing the hydrolysis time. This stepwise effect was expected since TFA disrupts glycosidic bonds selectively, at different rates depending on the linked monosaccharide [46]. For example, the disruption of bonds from amino sugars takes a longer time, while pentoses are among the first monosaccharides to be released and degraded under longer reaction times (>2 h).

**Figure 2:**
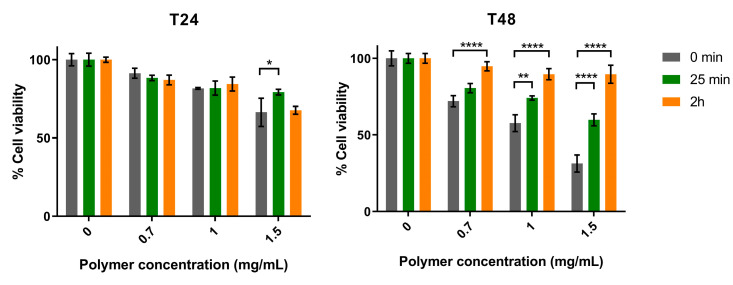
Effect of the *Synechocystis* Δ*sigF* polymer (0 min), and its variants obtained after hydrolysis with TFA for 25 min or 2 h, on the viability of human melanoma (Mewo) cell line evaluated with the PrestoBlue^TM^ viability assay. Cells were treated with 0.7, 1 or 1.5 mg/mL of polymer for 24 or 48 h (T24 and T48, respectively). Cells were also treated with polymer vehicle as control, showing no differences to Blank *(data not shown)*. Results are expressed in relation to Blank and are represented as mean ± STD of three independent experiments (* *p* < 0.05, ** *p* < 0.01 and **** *p* < 0.0001).

Subsequently, the effect of two polymer variants (obtained after 25 min and 2 h of TFA hydrolysis) on the viability of Mewo cells was evaluated. Overall, our results showed that both variants had less antitumor activity than the original Δ*sigF* polymer, and that after 48 h of treatment there was a significant decrease in bioactivity for the three polymer concentrations tested (Figure 2) [33]. Moreover, the variant with lower abundance of high MM fractions (2 h hydrolysis) had the lowest effect on Mewo cells viability, indicating that the high MM fractions play an important role in its antitumor activity. 

One should bear in mind that although hydrolysis with TFA is considered mild and believed not to cause significant desulfation, this effect cannot be completely ruled out [47,48]. Considering this, the Δ*sigF* polymer was also subjected to hydrolysis with HCl, which led to the formation of polymer variants mainly composed by low MM fractions (<342.30 Da) (Figure S1). As expected, the disruption of glycosidic bonds by HCl was quite fast, and HCl had a stronger action than TFA. The polymer variants obtained not only had alterations in their MM distribution, but also differed in sulfate content (Figure S2), being the complete desulfation achieved after 2 h of HCl hydrolysis. This is in agreement with the saturation point described for the HCl hydrolytic reaction but, as previously reported, the degradation of some sugars such as uronic acids might also occur [49,50]. The polymer variants obtained after HCl hydrolysis were also tested regarding their antitumor potential in Mewo cells. The results were similar to those obtained with the TFA variants, with the HCl variants having the same or lower antitumor activity compared to the original polymer (Figure S3). Altogether, these results indicate that hydrolysis and/or removal of sulfate groups decrease the bioactivity of the polymer.

This is in accordance with a previous study that reported that the hydrolysis of the EPS from *Synechocystis aquatilis* with TFA led to the loss of its bioactivity as inhibitor of the human complement system [47]. Furthermore, recent studies showed that hydrolyzed fucoidans from different sources maintained or reduced their antitumor activity compared to the original polymer [44]. On the other hand, our findings do not support the hypothesis that polymer variants with lower MM fractions could have higher antitumor activity by being more easily internalized by the cells [43,51].

### 3.2. Reduction of Peptide Content and Evaluation of the Antitumor Activity In Vitro

**Figure 3:**
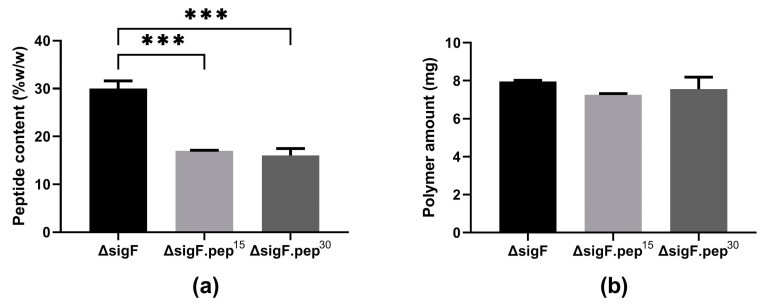
(**a**) Peptide content and (**b**) amount of *Synechocystis* Δ*sigF* lyophilized polymer before (Δ*sigF*) and after trichloroacetic acid (TCA) treatment for 15 (Δ*sigF*.pep15) or 30 min (Δ*sigF*.pep30). Peptide content was quantified by the Lowry method. Results are represented as mean ± STD of three technical replicates (*** *p* ≤ 0.001).

To evaluate the impact of peptide content on the antitumor activity of *Synechocystis* Δ*sigF* polymer (~27% *w/w* polymer dry weight, [27]), an aqueous solution of the polymer was incubated for 15 or 30 min with trichloroacetic acid (TCA). After either 15 or 30 min, the amount of peptides was reduced by approximately 40% (Figure 3a), with a minimal loss (~5%) in the yield of the polymer (Figure 3b) [36].

Although TCA precipitation is usually highly efficient and widely used to remove peptides from polysaccharides [52,53,54], the efficiency reported here is lower than previously reported for polysaccharidic polymers from heterotrophic bacterial and plant sources [55,56], but in the same range as the ones reported for other cyanobacterial polymers [57,58]. In some cases, such as for polysaccharides from *Spirulina platensis*, higher TCA concentrations had to be used to achieve a precipitation efficiency similar to the one reported here (~41% less peptides) [57]. These differences can be attributed to the high complexity of the cyanobacterial polymers and/or to a stronger association between the peptides and the polymer. Nevertheless, the amount of Δ*sigF* polymer that was lost during the TCA treatment in our study was among the lowest reported, even when compared with similar treatments using polymers from non-cyanobacterial sources [55]. Since the decrease in peptide content was similar in the two treatments (Figure 3a), the polymer variant obtained after 15 min (Δ*sigF*.pep-) was the one selected to proceed. 

**Figure 4:**
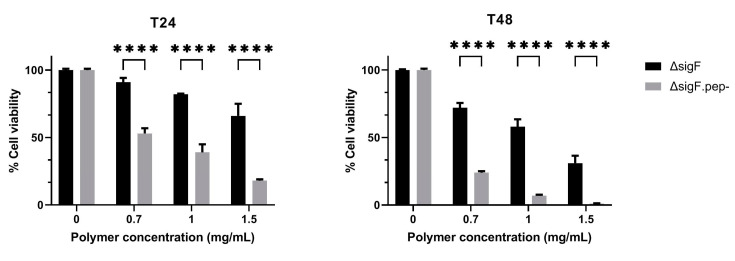
Effect of the *Synechocystis* Δ*sigF* polymer (Δ*sigF*) and Δ*sigF* polymer with reduced peptide content (Δ*sigF*.pep-) on the viability of human melanoma (Mewo) cell line, analyzed using PrestoBlue™ viability assay. Cells were treated with 0.7, 1 or 1.5 mg/mL of polymer for 24 or 48 h (T24 and T48, respectively). Cell treatment with polymer vehicle showed no differences compared to Blank (data not shown). Results are express in relation to Blank and are represented as mean ± STD of three independent experiments (**** *p* ≤ 0.0001).

The antitumor potential of Δ*sigF*.pep- variant was tested *in vitro* in Mewo cells and compared to the Δ*sigF* polymer with intact peptide content (Figure 4) [36]. Both polymers induced a strong decrease in cells viability in a time- and dose-dependent manner being this effect significantly stronger following treatment with the Δ*sigF*.pep- variant compared to the treatment with the original Δ*sigF* (Figure 4). At the lowest concentration tested (0.7 mg/mL), the Δ*sigF* polymer reduced Mewo cells viability in approximately 9% and 28% after 24 h and 48 h of treatment respectively, while the cells treated with Δ*sigF*.pep- showed a viability decrease of about 47% and 76%, respectively.

This is in line with the results obtained by Garbacki et al. [59], which showed that TCA peptide precipitation from isolated cyanobacterial capsular polysaccharides (CPS) from two *Phormidium* strains increased the anti-inflammatory activity of these polymers in ~60%. One possible explanation for the stronger bioactivity of the Δ*sigF*.pep- compared to Δ*sigF* may be the increased exposure of bioactive component(s). However, in our study we cannot exclude that at least some of the bioactive components could be the remaining peptides, since only a partial peptide removal was achieved with TCA treatment. A previous work reported that specific protein fractions in the EPS from the cyanobacterium *Gloeocapsa* sp. might be involved in their antitumor activity towards human cervical carcinoma (HeLa) cells [21]. Furthermore, other studies have shown that peptides present in the EPS from microalgae are important for their biological activity [60,61,62], namely a glycoprotein found in the EPS from *Chlorella vulgaris* that was associated with their antitumor activity [63].

### 3.3. Assessment of the Antitumor Activity In Vivo

To validate *in vivo* the antitumor potential of our original polymer (Δ*sigF*) and its most promising variant (Δ*sigF*.pep-), the chick embryo chorioallantoic membrane (CAM) assay was used. This is a low-cost, reproducible, and reliable preclinical cancer model that allows investigation of tumor growth and angiogenesis *in vivo*, being often used to evaluate potential anticancer drugs [64]. Therefore, and following the *in vitro* results showing that the variant Δ*sigF*.pep- had stronger antitumor potential, CAM assays were performed [35]. For this purpose, CAMs were inoculated with Mewo cells at embryonic development day 9 (EDD9), together with the Δ*sigF* polymer, its Δ*sigF*.pep- variant or with their vehicle (DMEM, control). The lowest concentration (0.7 mg/mL) was chosen for this analysis. A dose–response curve for the effect of the Δ*sigF*.pep- variant in Mewo cells is shown in Figure S4, while for the Δ*sigF* polymer it has been previously published in [20].

**Figure 5:**
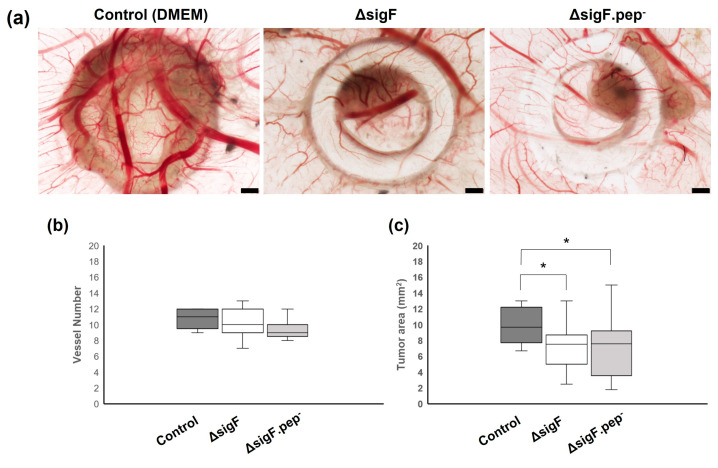
Effect of the polymers analyzed by the chick embryo chorioallantoic membrane (CAM) assay. Mewo cells were inoculated on the CAM at embryonic development day 9 (EDD9) with polymers Δ*sigF* and Δ*sigF*.pep-, or with vehicle solvent (DMEM). At EDD13, CAM and respective tumors were analyzed. (**a**) Representative *ex ovo* images showing the ring used for cell inoculation, the tumor formed and blood vessels. Images are 20× magnification and scale bar = 500 µm. (**b**) Angiogenic analysis. Quantification of the number of vessels (<20 µm) growing towards the inoculation site, delimited by the ring mark, induced by vehicle solvent (Control n = 9, Δ*sigF* n = 19; Δ*sigF*.pep- n = 13). (**c**) Tumor growth analysis of CAM xenografted tumors area measured in mm^2^ (Control n = 13, Δ*sigF* n = 18; Δ*sigF*.pep- n = 17) (* *p* < 0.05).

After four days of treatment, CAMs were retrieved and analyzed *ex ovo* to evaluate vessel formation (angiogenesis) and tumor growth (Figure 5). Results regarding the number of blood vessels counted on the region of interest showed no significant differences between polymer and control treatments, although a tendency for a slight decrease in vessel count was observed with Δ*sigF*.pep- variant, compared to the control (9.5 ± 1.2 and 10.7 ± 1.2, respectively) (Figure 5b). On the other hand, evaluation of tumor size showed that treatment with both polymers (Δ*sigF* polymer and Δ*sigF*.pep- variant) resulted in a significant decrease of tumor area (7.2 ± 2.1 mm^2^ and 7.1± 3.7 mm^2^, respectively) compared to the control condition (9.9 ± 2.1 mm^2^) (Figure 5c). These results, demonstrating *in vivo* antitumor activity of both polymers, are in accordance with previously *in vitro* results (Figure 4). However, no significant differences were observed when comparing the effect of the two polymers on tumor growth *in vivo*.

**Figure 6:**
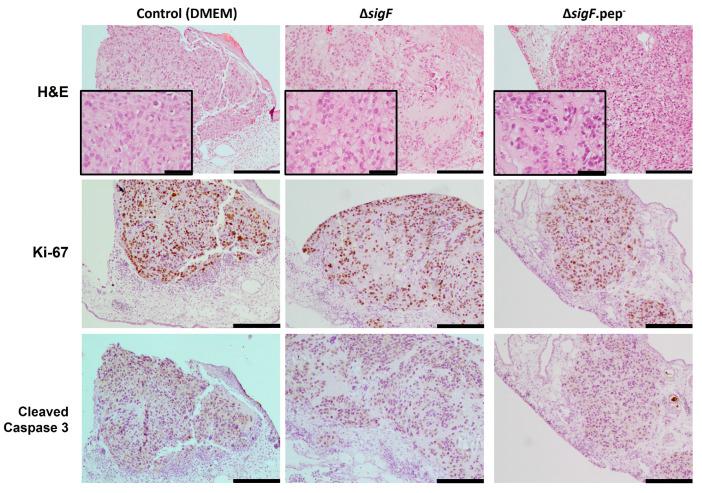
Representative images of the excised tumor xenografts from CAM after treatment with polymers Δ*sigF* and Δ*sigF*.pep-, or with vehicle solvent (DMEM), stained with H&E, and immunostained for Ki-67 and cleaved caspase-3. Scale bar = 200 μm (except in the insets of H&E, in which scale bar = 50 μm).

To gain insight into potential mechanisms involved in the effect of these polymers in tumor growth, CAM xenografted tumors were further evaluated histologically. Hematoxylin-eosin staining showed differences in tumor cell morphology (Figure 6). While the control tumors (treated with DMEM) exhibited a more compact and cellular structure, the tumors treated with the polymers (in particular with Δ*sigF* polymer) were less compact (with more stroma and less cells within the same area). Since we previously showed that short term exposition to the Δ*sigF* polymer increased apoptosis markers [20], here we proceed to evaluate proliferation and apoptosis in the CAM tumors. No differences were observed regarding the percentage of Ki-67-stained cells per slide, with polymer treated tumors presenting similar levels compared to controls (Δ*sigF*: 70.3 ± 4%; Δ*sigF*.pep-: 71.9 ± 4% Control: 73.8 ± 2%, Figure 6 middle panel). Moreover, no evident differences in caspase 3 cleavage were observed in any of the conditions (Figure 6 lower panel). We cannot exclude the possibility that alterations in apoptosis (or proliferation, for that matter) may be occurring in polymer-treated cells at earlier time points. It is possible that the observed differences in tumor cell morphology may result from an initial effect of the polymers on the inoculated cells, which was not evident in the proliferative or apoptotic profiles of the surviving cells at EDD13. In addition, in our previous study [20], the experiments were carried out only in *in vitro* conditions and using different methodologies (Annexin V/PI staining by flow cytometry and cleaved caspase-3 by Western blot). Finally, it is important to consider that the use of different models (*in vitro* vs. *in vivo*) may also impact the results, as *in vitro* experiments do not take into account the complex interactions between tumor cells and the surrounding stroma.

## 4. Conclusions

Overall, *Synechocystis* Δ*sigF* polymer was shown to be a powerful platform to develop polymer variants with *in vitro* and *in vivo* antitumor activity. We demonstrated that high molecular mass fractions of the Δ*sigF* polymer were important for its antitumor activity in human melanoma cells, and that the reduction of the peptide content generated a polymer variant with enhanced *in vitro* antitumor activity. Importantly, the antitumor potential of Δ*sigF* polymer and its Δ*sigF*.pep- variant was validated *in vivo*, with both polymers significantly decreasing xenografted CAM tumor growth and affecting tumor morphology, by promoting less compact tumors. Further studies are necessary to fully disclose the mechanisms behind the effects of these polymers in tumor growth, as well as to overcome technical issues usually associated with polymers with high molecular mass fractions/viscosity. Nevertheless, the methodologies herein implemented represent straightforward strategies for the design and testing of tailored cyanobacterial extracellular polymers for biotechnological/biomedical applications. Moreover, this work further strengthens the relevance of evaluating the antitumor potential of this type of natural polymers both *in vitro* and *in vivo*.

## Data Availability

Not applicable.

## Supplementary Materials

The following supporting information can be downloaded at: https://www.mdpi.com/article/10.3390/polym15061382/s1, Figure S1: Molecular mass distribution of *Synechocystis* sp. PCC 6803 Δ*sigF* polymer before and after hydrolysis with HCl for different time periods; Figure S2: Quantification of the sulfate released from *Synechocystis* Δ*sigF* polymer after HCl hydrolysis (from 10 min to 2 h), and the percentage of desulfation obtained; Figure S3: Effect of the *Synechocystis* Δ*sigF* polymer, and its variants obtained after hydrolysis with HCl (from 10 min to 2 h), on the viability of human melanoma (Mewo) cell line evaluated with the PrestoBlue^TM^ viability assay; Figure S4: Effect of the *Synechocystis* Δ*sigF* polymer with reduced peptide content (Δ*sigF*.pep-) on the viability of human melanoma (Mewo) cell line, analyzed using the PrestoBlue™ viability assay.
