# Supplementary material for: Assessing the Antitumor Potential of Variants of the Extracellular Carbohydrate Polymer from Synechocystis ΔsigF Mutant
Source: Polymers (Basel). 2023 Mar 10;15(6):1382. doi: 10.3390/polym15061382 (PMC10057057; doi:10.3390/polym15061382)
Supplement: Supplementary file 1 [file polymers-15-01382-s001.zip › polymers-2249349-SI/Mota et al 2023_Polymers_Suppl material.pdf]

polymers

MDPI

Supplementary Materials

Assessing the antitumor potential of variants of the extracellular carbohydrate polymer from *Synechocystis* Δ*sigF* mutant

Rita Mota [1,2,#], Raquel T. Lima [1,3,4], Carlos Flores [1,2,5,§], Juliana F. Silva [1,2,6], Beatriz Cruz [1,2,6], Bárbara Alves [1,3,7], Marta T. Pinto [1,3], Alessandra Adessi [8], Sara B. Pereira [1,2], Roberto De Philippis [8], Paula Soares [1,3,4] and Paula Tamagnini [1,2,6*]

[1] i3S - Instituto de Investigação e Inovação em Saúde, Universidade do Porto, Rua Alfredo Allen 208, 4200-135 Porto, Portugal
[2] IBMC - Instituto de Biologia Molecular e Celular, Universidade do Porto, Rua Alfredo Allen 208, 4200-135 Porto, Portugal
[3] IPATIMUP - Institute of Molecular Pathology and Immunology of the University of Porto, Rua Alfredo Allen 208, 4200-135 Porto, Portugal
[4] FMUP - Department of Pathology, Faculty of Medicine, University of Porto, Alameda Prof. Hernâni Monteiro, 4200-319 Porto, Portugal
[5] ICBAS - Instituto de Ciências Biomédicas Abel Salazar, Universidade do Porto, Rua de Jorge Viterbo Ferreira 228, 4050-313 Porto, Portugal
[6] FCUP - Departamento de Biologia, Faculdade de Ciências, Universidade do Porto, Rua do Campo Alegre, s/n, 4169-007 Porto, Portugal
[7] School of Allied Health Sciences of Polytechnic Institute of Porto, Rua Dr. António Bernardino de Almeida 400, 4200-072 Porto, Portugal
[8] DAGRI - Department of Agriculture, Food, Environment and Forestry, University of Florence, Via Maragliano 77, 50144 Firenze, Italy
[#] Rita Mota current affiliation: Austrian Centre of Industrial Biotechnology (ACIB), Konrad-Lorenz-Straße 20, 3430 Tulln, Austria
[§] Carlos Flores current affiliation: Centre for Urological Biology, Division of Medicine, University College London (UCL), Royal Free Hospital Campus, Rowland Hill Street NW3 2PF, London, United Kingdom

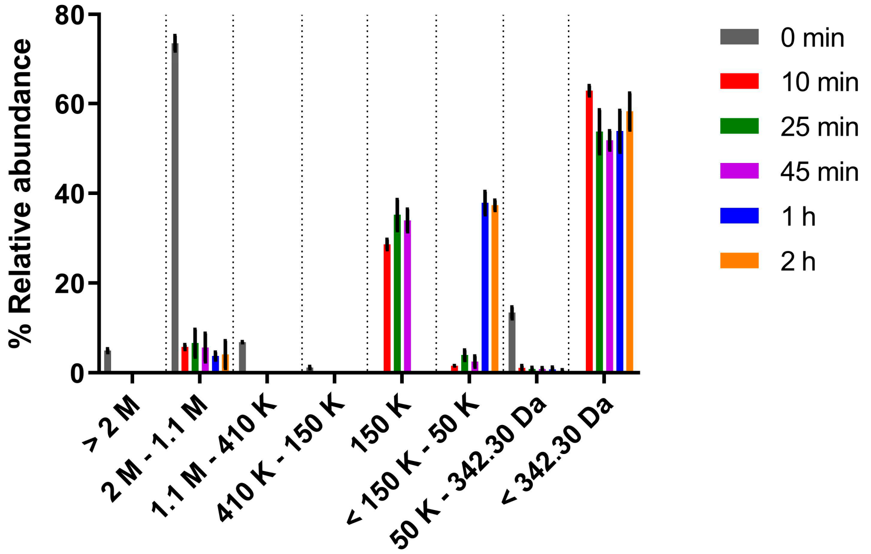

Figure S1. Molecular mass distribution of *Synechocystis* Δ*sigF* polymer before and after hydrolysis with HCl for different time periods. Measurements were made using size exclusion chromatography (SEC). Results are represented as mean ± STD of three biological and three technical replicates.

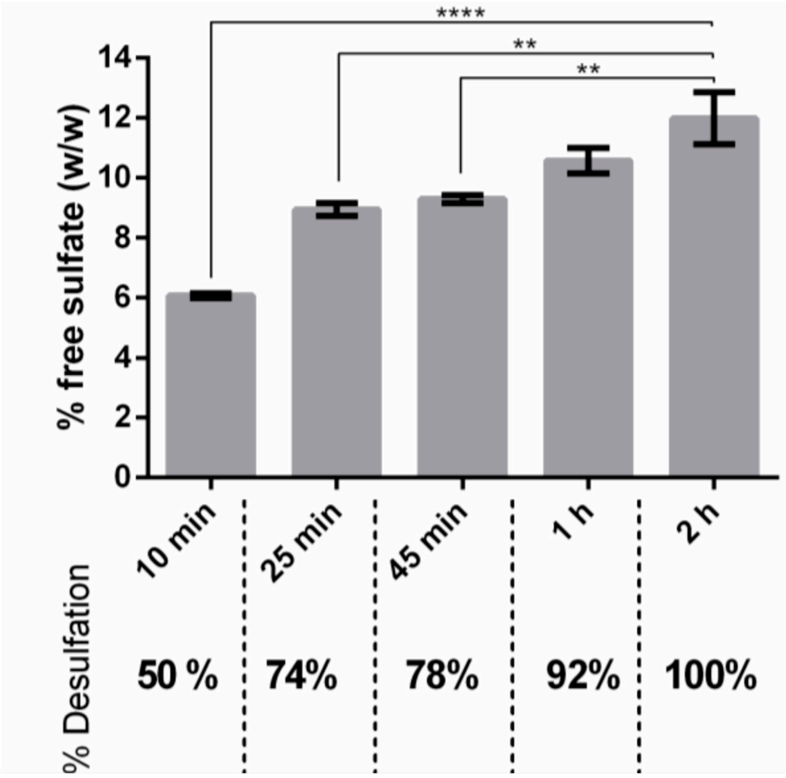

Figure S2. Quantification of the sulfate released from *Synechocystis* Δ*sigF* polymer after HCl hydrolysis (from 10 min to 2 h), and the percentage of desulfation obtained. Statistical analysis is presented in comparison to the maximum time of hydrolysis (** $p \leq 0.01$; **** $p \leq 0.0001$).

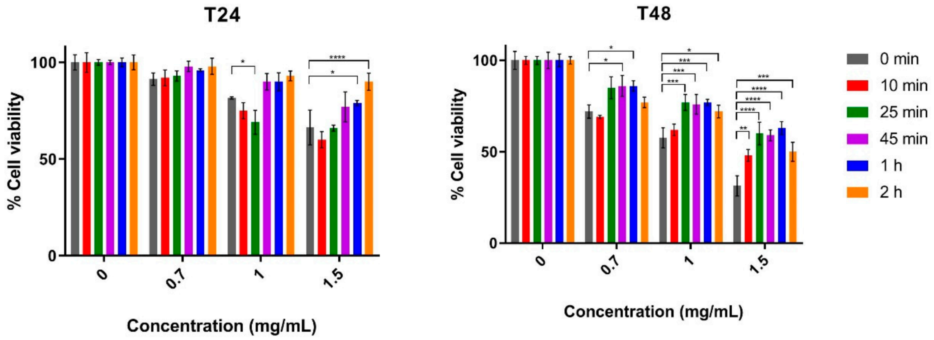

Figure S3. Effect of the *Synechocystis* Δ*sigF* polymer (0 min), and its variants obtained after hydrolysis with HCl (from 10 min to 2 h), on the viability of human melanoma (Mewo) cell line evaluated with the PrestoBlue™ viability assay. Cells were treated with 0.7, 1 or 1.5 mg/mL of polymer for 24 or 48 h (T24 and T48, respectively). Cells were also treated with polymer vehicle as control, showing no differences to Blank (data not shown). Results are expressed in relation to Blank and are represented as mean ± STD of three independent experiments (* $p < 0.05$, ** $p < 0.01$ *** $p \leq 0.001$; and **** $p < 0.0001$).

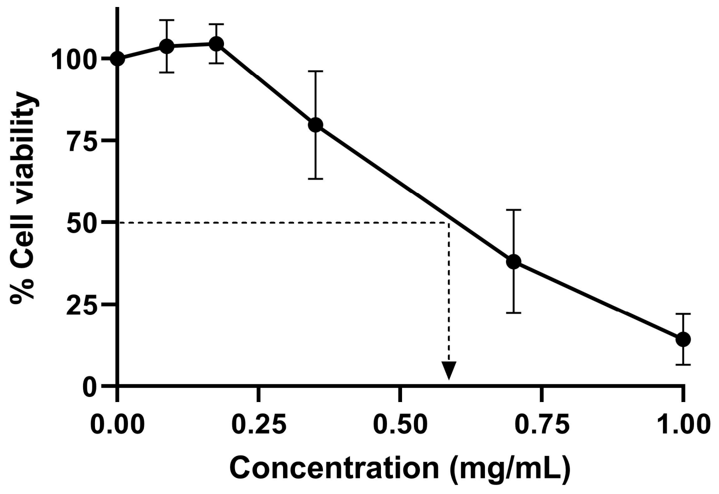

Figure S4. Effect of the *Synechocystis* Δ*sigF* polymer with reduced peptide content (Δ*sigF*.pep-) on the viability of human melanoma (Mewo) cell line, analyzed using the PrestoBlue™ viability assay. Cells were treated with increasing concentrations of the polymer for 48 h. Cells treated with polymer vehicle were used as controls showing no differences to Blank (data not shown). Results are express in relation to Blank and are represented as mean ± STD of four independent experiments.
